# Supplementary material for: Predicting visual function by interpreting a neuronal wiring diagram
Source: Nature. 2024 Oct 2;634(8032):113–23. doi: 10.1038/s41586-024-07953-5 (PMC11446822; doi:10.1038/s41586-024-07953-5)
Supplement: Supplementary file 6 — CRF and ERF predictions for individual TmY4 and TmY9 cells. Analogous to Supplementary Data 3, but for TmY target types. Shown are the top four monosynaptic pathways, the strongest pathway passing through each of the top ten intermediary types (ranking from Extended Data Fig. 7), and the trisynaptic pathway Tm1–TmY–Dm3–TmY (see the section entitled Prediction of spatial normalization). [file 41586_2024_7953_MOESM6_ESM.zip › DataS4/TmY9q/720575940637948742.pdf]

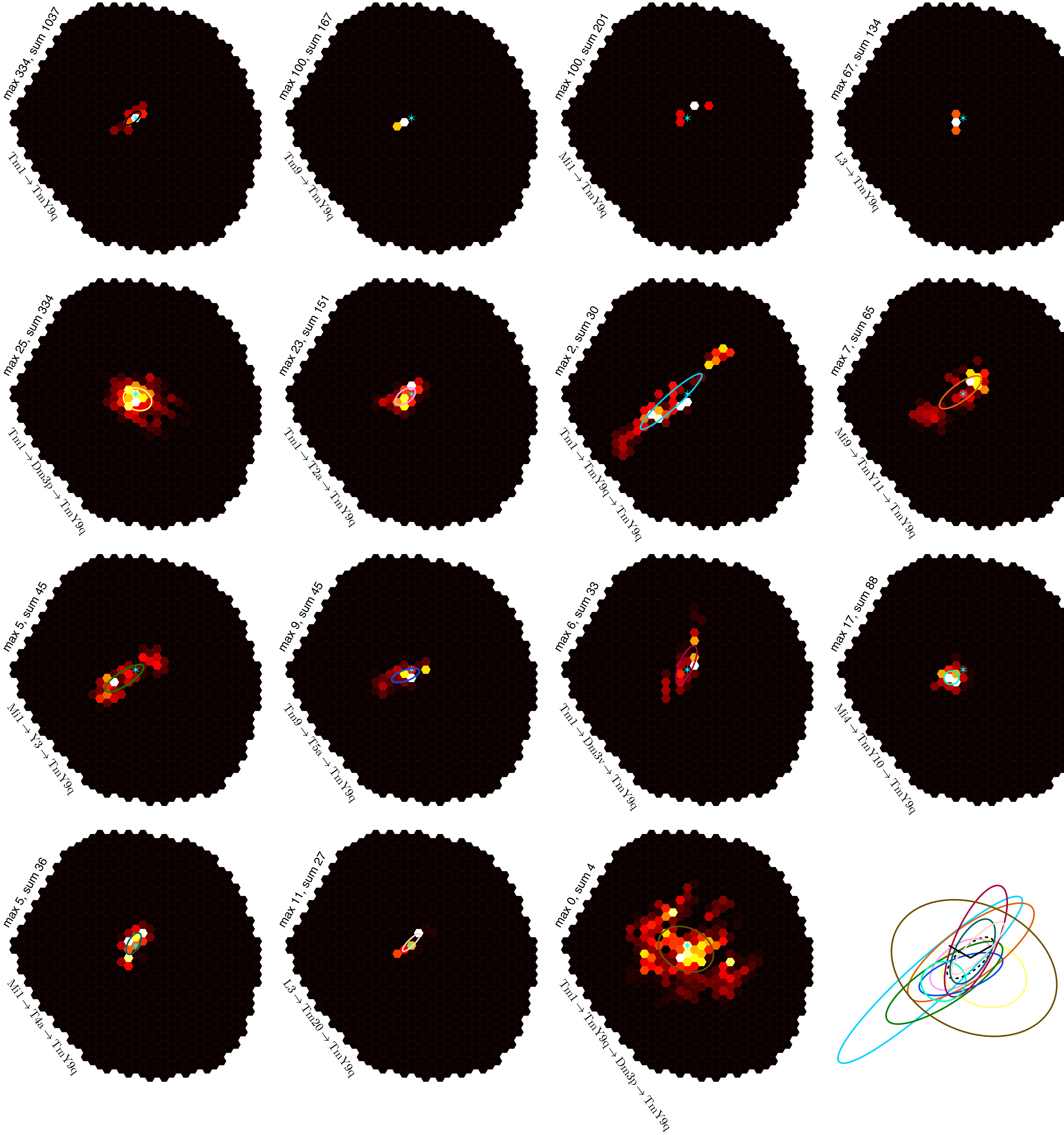
max 334, sum 1037
Tm1 → TmY9q
max 100, sum 167
Tm9 → TmY9q
max 100, sum 201
Mi1 → TmY9q
max 67, sum 134
L3 → TmY9q
max 25, sum 334
Tm1 → Dm3p → TmY9q
max 23, sum 151
Tm1 → T2a → TmY9q
max 2, sum 30
Tm1 → TmY9q → TmY9q
max 7, sum 65
Mi9 → TmY11 → TmY9q
max 5, sum 45
Mi1 → Y3 → TmY9q
max 9, sum 45
Tm9 → T5a → TmY9q
max 6, sum 33
Tm1 → Dm3v → TmY9q
max 17, sum 88
Mi4 → TmY10 → TmY9q
max 5, sum 36
Mi1 → T4a → TmY9q
max 11, sum 27
L3 → Tm20 → TmY9q
max 0, sum 4
Tm1 → TmY9q → Dm3p → TmY9q
